# Fabrication and Integration of Functionalized N-rGO-Ni/Ag and N-rGO-Ni/Co Nanocomposites as Synergistic Oxygen Electrocatalysts in Fuel Cells

**DOI:** 10.3390/nano12040585

**Published:** 2022-02-09

**Authors:** Muhammad Arif, Salma Bilal, Anwar ul Haq Ali Shah

**Affiliations:** 1 National Centre of Excellence in Physical Chemistry, University of Peshawar, Peshawar 25120, Pakistan; arifchem46@yahoo.com; 2 Institute of Chemical Sciences, University of Peshawar, Peshawar 25120, Pakistan; anwarulhaqalishah@uop.edu.pk

**Keywords:** N-doped reduced graphene oxide, oxygen reduction reaction, oxygen evolution reaction, bi-functional electrocatalysis

## Abstract

Fabrication of composites by developing simple techniques can be an efficient way to modify the desire properties of the materials. This paper presents a detailed study on synthesis of low cost and efficient nitrogen doped reduced graphene oxide nickle-silver (N-rGO-Ni/Ag) and nickel-cobalt (N-rGO-Ni/Co) nanocomposites as electrocatalysts in fuel cell using one-pot blended reflux condensation route. An admirable correlation in the structures and properties of the synthesized nanocomposites was observed. The Oxygen Reduction Reaction (ORR) values for N-rGO-Ni/Ag and N-rGO-Ni/Co calculated from the onset potential, using Linear Sweep Voltammetry (LSV), were found to be 1.096 and 1.146. While the half wave potential were determined to be 1.046 and 1.106, respectively, N-rGO-Ni/Ag and N-rGO-Ni/Co. The Tafel and bi-functional (ORR/OER) values were calculated as 76 and 35 mV/decade and 1.23 and 1.12 V, respectively, for N-rGO-Ni/Ag and N-rGO-Ni/Co. The lower onset and half wave potential, low charge transfer resistance (R_ct_ = 1.20 Ω/cm^2^) and internal solution resistance (R_s_ = 8.84 × 10^−1^ Ω/cm^2^), lower Tafel values (35 mV), satisfactory LSV measurements and mass activity (24.5 at 1.056 V for ORR and 54.9 at 1.056 for OER) demonstrate the remarkable electrocatalytic activity of N-rGO-Ni/Co for both ORR and OER. The chronamperometric stability for synthesized nanocomposites was found satisfactory up to 10 h.

## 1. Introduction

The alarming rate of ever-increasing energy demand stimulated academics’ and researchers’ attention towards state-of-the-art energy systems. Fuel cells are the next-generation renewable energy devices. They offer the prospect of a high efficiency power source for vehicles, stationary power generation, and portable power devices etc. [1]. Fuel cells basically rely on oxygen reduction reaction (ORR) and oxygen evolution reaction (OER) [2], where catalysts are at the heart for sluggish kinetics of ORR and OER processes. To overcome the slothful kinetics of ORR and OER, different metal catalysts were employed as electrode materials. To date, Pt/C, iridium- (Ir), and ruthenium (Ru)-based catalysts are the mainstay, where the scarcity and high costs are the bottleneck for commercialization and applications. Consequently, the researchers’ interest was drawn toward inexpensive and readily accessible transition metals (TMs) which could be assigned as a good alternative for high-cost Pt, Ir, and Ru metal-based electrocatalysts. These metals were often put to use in catalysis in fuel cells in the form of transitional metal complexes such as chalcogenides and metal oxides [3].

In recent years graphene-transition metals nanocomposites doped with heteroatoms such as B, N, P, and S attracted immense interest of researchers of new designs of hybrid materials for various applications. These hybrids were used in electronics [4], batteries [5], fuel cells [6], and other energy storage devices [7]. The heteroatom doping within graphene structure causes charge distribution and provides marked active sites which manifest a marvelous potential as ORR and OER electrocatalysts. Furthermore, the dual and ternary doped graphitic carbon further exploits the synergic effects for better ORR/OER catalytic activity. To develop low cost and efficient catalysts as electrode materials, different efforts were made. Hong Zhao et al. wrapped Fe_2_O_3_ in dual doped phosphorous and sulfur graphitic which represent good response for both ORR and OER [5]. Recently, Askari and his group developed binary transition metal oxide nanocomposite (MnNi_2_O_4_/rGO) followed by a hydrothermal method [8]. The resultant nanocomposite exhibited superior electrocatalytic properties. In this connection, Chen and his group fabricated high performance nitrogen doped graphene supported by iron and molybdenum (FeMo-NG) as electrocatalysts for oxygen reduction reactions (ORR) with favorable kinetics [9]. Similarly, Vincenza et al. investigated Fe_2_O_3_/rGO, CoO/rGO, and CoFe_2_O_4_/rGO nanocomposites for electrocatalytic determination in fuel cells [10]. In the similar way, Hanwen Xu and his group explored the catalytic properties of 2D MoS_2_ based materials for ORR [11]. Hangil Lee asserted the enhanced catalytic activity of rGO by the addition of various transiton metals (Cr, Fe, Co) [12]. Likewise, Yexin Dai et al. fabricated transitional metals (Ni, Co, Fe) reduced graphene oxide nanocomposites doped with nitrogen (N) and sulfur (S) for possible applications in fuel cells [13].

The present study aims to fabricate nitrogen doped functionalized reduced graphene oxide (N-rGO) nanocomposites, supported with nickel (Ni), cobalt (Co), and silver (Ag) for possible applications in fuel cells. The strong affinity of the free functionalities of hydroxyl and carboxylic group of reduced graphene oxide tend to complex Ni, Co, and Ag cations. This tendency crafts the strong interaction within N-rGO-Ni/Ag and N-rGO-Ni/Co composite systems, verified by FTIR and XPS analysis. The fabricated nanocomposites were further characterized by different physico-chemical techniques. However, the special emphasis was given to electrochemical studies for possible applications in fuel cells based on ORR and OER processes.

## 2. Materials and Methods

The experimental section is broadly divided into two phases. The first phase comprises the synthesis of reduced graphene oxide doped with nitrogen (N-rGO), while the second phase covers the synthesis of N-rGO-Ni/Ag and N-rGO-Ni/Co nanocomposites.

### 2.1. Synthesis of Nitrogen-Doped Reduced Graphene Oxide (N-rGO)

Graphene oxide (GO) was synthesized followed by modified Hummer’s method [14]. In order to synthesize hetroatom-doped reduced graphene oxide, nitrogen was used as a doping agent. The reduction and doping of as synthesized graphene oxide was carried out using one pot blending technique. In a typical procedure, 35 mg of graphene oxide was dispersed in 70 mL deionized water via ultrasonication in nitrogen atmosphere for 3 h, furnishing a homogeneous colloidal dispersion. Five microliters of hydrazine hydrate was added to the dispersion and transfer into the teflon lined hydrothermal autoclave at 180 °C for 12 h. Subsequently, the reaction was stopped by quenched into cold water. The product obtained was washed several times with DD water and ethanol. Finally, the product was dried at room temperature for three days and stored prior to further process.

### 2.2. Synthesis of Nitrogen-Doped Reduced Graphene Oxide-Nickel/Sliver (N-rGO-Ni/Ag) Nanocomposite

**Scheme 1:**
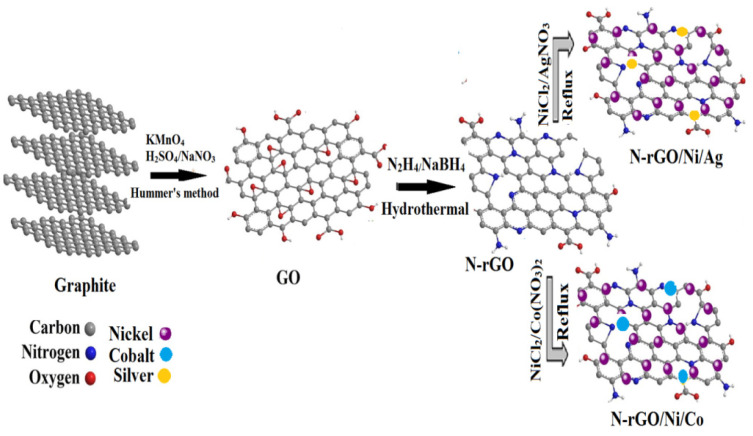
The schematic representation of the active sites and free functionalities of GO for counter part of metal precursor.

The synthesized N-doped reduced graphene oxide was dispersed in 60 mL deionized water to make homogenous solution. In the same way, 20 mL of 0.052 M NiCl_2_ and 0.045 M AgNO_3_ precursors were added to the suspension. After a while 0.375 M of NaOH was added drop wise till the pH of the solution reached 10.5. The as prepared mixture was refluxed at 120 °C for 3 h under nitrogen atmosphere. The mixture was calcined at 600 °C for 6 h, and then washed several times with DD water and ethanol followed by centrifugation at 6000 rpm. Ultimately, the synthesized N-rGO-Ni/Ag composite was dried at 50 °C for 2 h in a vacuum oven and stored for characterization [15]. The same procedure was followed for fabrication of N-rGO-Ni/Co nanocomposite using cobalt Co(NO_3_)_2_ and NiCl_2_ blend. The synthesis routs are mapped in Scheme 1 below.

### 2.3. Characterization

Fourier-transform infrared spectroscopy (FTIR) analysis of the materials was carried out in the range of 400–4000 cm^−1^ by using IRAffinity-1S Shimadzu Fourier Transform Infrared Spectrophotometer (Shimadzu, Tokyo, Japan). XRD (X-ray diffractometer) patterns were achieved by using Cu Kα radiations (λ = 1.5405 Å) JEOL JDX-3532 (JEOL, Tokyo, Japan). SEM (Scanning electron microscopy) and TEM (transmission electron microscopy) were done by electron microscope JSM-6490 and JEM-2100 (JEOL, Tokyo, Japan), respectively. BET/BJH measurements were accomplished through surface area analyzer model NOVA2200e Quantachrome (Boynton Beach, FL, USA), while the surface engineering ofthe materials were examined from XPS analysis (Model: Quantum 2000 Scanning SK Microprobe, Physical Electronics, Chanhassen, MN, USA). Electrochemical characterizations were carried out in three electrode assembly using Gamry’s potentiostat/galvanostat, Reference 3000 ZRA and Gamry’s Rotating Disk Electrode (RDE 710, Boynton Beach, FL, USA).

## 3. Results and Discussion

### 3.1. Fourier Transform Infrared Spectroscopy (FTIR)

**Figure 1:**
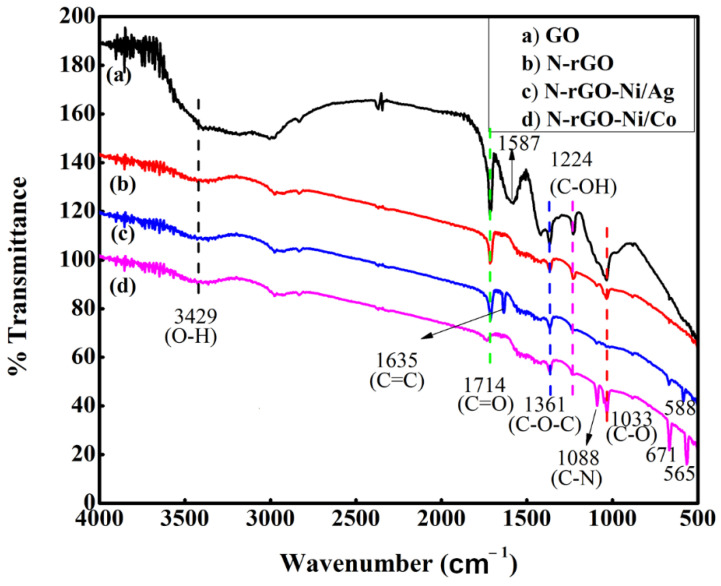
FTIR spectroscopy of (**a**) GO, (**b**) N-rGO, (**c**) N-rGO-Ni/Ag, and (**d**) N-rGO-Ni/Co.

FT-IR spectroscopy was performed in order to find the nature of GO and its complexation in composite systems. The broad absorption peaks at about 3429 cm^−1^ represent the stretching vibration of O-H from water contents in the GO sheet. The major band positioned at about 1714 cm^−1^ is assigned to C=O stretching vibration. The bands at about 1361 cm^−1^, 1224 cm^−1^, and 1033 cm^−1^ are associated with carboxy C–O, epoxy C–O and alkoxy C–O–C respectively [16]. The characteristics peak intensities at 1714 cm^−1^, 1361 cm^−1^ and 1224 cm^−1^ shown in Figure 1 are decreased, which indicates the reduction of GO. Likewise, the band at 1587 cm^−1^ is vanished in case of N-rGO and its composites due to the strong reduction effect, verifying the reduction of GO. Furthermore, the peaks in all the spectra at about 1088 and 1033 cm^−1^ are credited to C–N and C–O–C stretching vibration, respectively. On the other hand, the peaks at 671,588 and 565 cm^−1^ are assigned to stretching vibrations of Ni–OAg–O and Co–O bonds in the GO sheet in that order. The extra peak at 1635 cm^−1^ in the spectrum of N-rGO-Ni/Ag can be inferred as O–H in water [17,18,19]. These illustrations verify the successful synthesis, reduction, N doping and complexation of GO.

### 3.2. X-ray Diffraction Analysis (XRD)

**Figure 2:**
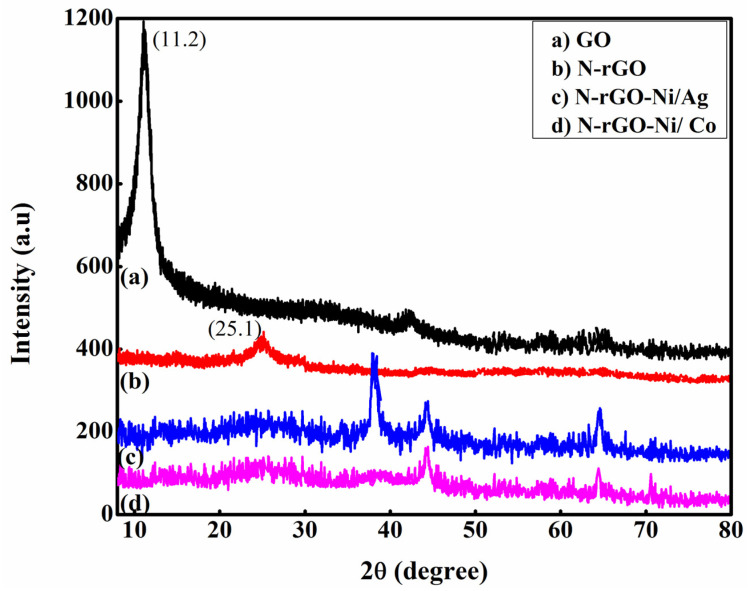
X-ray diffraction analysis of (**a**) GO, (**b**) N-rGO, (**c**) N-rGO-Ni/Ag, and (**d**) N-rGO-Ni/Co.

The XRD patterns of synthesized materials are depicted in Figure 2. The pristine GO shows atypical peak at 2θ = 11.2° corresponding to (002) plane with interplanar spacing of 0.806 nm. The peak position is shifted dramatically to 2θ = 25.16° in case of N-rGO, which has interplanar spacing of 0.353 nm. The d-spacing comparison suggests that significant de-oxygenation occurred in pristine GO to produced graphene [20,21]. Similarly, the diffraction angles of N-rGO-Ni/Ag agreed well with the cubic crystal structure of nickel with metallic silver shown in Figure 2c. The peaks at 38.11°, 44.3 and 64.5° 2θ matched with JCPDS card No-73-1523, which is indexed for cubic Ni or NiO. The sharp diffraction peak for GO is absent in N-rGO-Ni/Ag hybrid but a small peak appears at 34.1°, attributed to high degree exfoliation of the graphene sheet in N-rGO-Ni/Ag hybrid. Moreover, the peak at 70.5° 2θ corresponds for Ni-Ag hybrid and signifies that Ni and Ag cations are fully merged within the GO matrix. In the similar way most of the peaks of N-rGO-Ni/Co composite are in close agreement with N-rGO-Ni/Ag, whereas the extra peak at 70.3° corresponds to the Ni-Co hybrid in the composite system. These illustrations suggest the structural feature and phase distribution of true and high-quality hybrid composite systems [22,23]. The average crystallite size was found to be 4.593 nm using Scherer’s equation

(1) $$D=\frac{K\lambda }{\beta \cos\theta }$$

where, D is the crystallites size (nm), K is the Scherer’s constant (0.9), λ is the wavelength of X-ray sources (0.15406 nm), β is the FWHM (radians) and θ is the peak position (radians).

### 3.3. Scanning Electron Microscopy (SEM)

**Figure 3:**
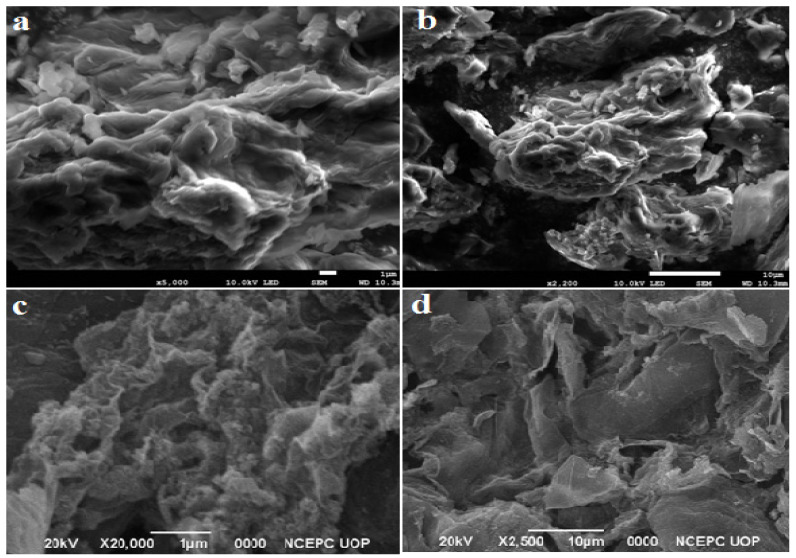
Scanning Electron Micrographs of (**a**) GO, (**b**) N-rGO, (**c**) N-rGO-Ni/Ag and (**d**) N-rGO-Ni/Co.

The micrograph in Figure 3a signifies that GO is made up of flaky texture, reflecting its layered microstructure with some cavities which are stacked and crumpled with each other. The larger interspaces of the layers edges of GO can be observed in Figure 3a [24]. The image in Figure 3b verifies the formation of multilayered reduced graphene oxide (N-rGO) which has relatively close surface morphology and implies well separated flakes of uniform size with bent corners configuration [25]. Figure 3c,d shows the micrographs of N-rGO-Ni/Ag and N-rGO-Ni/Co, respectively. These composites demonstrate more wrinkled morphology in comparison to N-rGO. It is possible that the oxygen containing functional groups on the surface of N-rGO were entirely removed and have strong van der Waals forces between metal particles and graphene layers. This effect made the graphene structure curlier and more wrinkled, leading to the formation of lamella structure [26,27]. Nevertheless, the particles of Ni, Ag and Co seem completely compatible and are embedded within the graphene matrix.

### 3.4. Transmission Electron Microscopy (TEM)

**Figure 4:**
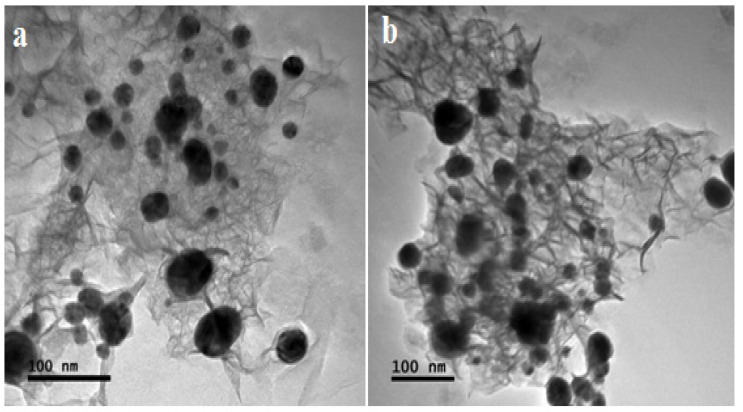
Transmission electron microscopy of (**a**) N-rGO-Ni-Ag and (**b**) N-rGO-Ni-Co.

Figure 4 presents the characteristic transmission electron micrographs of N-rGO-Ni/Ag and N-rGO-Ni/Co. The micrograph portrayal the transparent and thin carbon films of GO with an inter-layer distance roughly, 0.35 nm. The elastic corrugations and the scrolling edges may often effect the clarity of the layer structure of GO. However, our synthesized nanocomposites retain its transparency and reflect the homogeneous distributions and encapsulation of nanoparticles in the GO-TMs composite systems. The particles are almost spherical with average size of about 40–45 nm [28,29]. It is proposed that the electronegativity of oxygen atom of hydroxyl (–OH) and carboxyl (–COOH) in GO layer structure facilitated the orientation of the carbon cation radical. In addition, these groups build up the Vander wall interaction with Ni, Co and Ag particles. The TEM examinations of N-rGO-Ag-Ni and N-rGO-Ni-Co are in good agreement with reported literature [30].

### 3.5. BET and BJH Measurements

**Figure 5:**
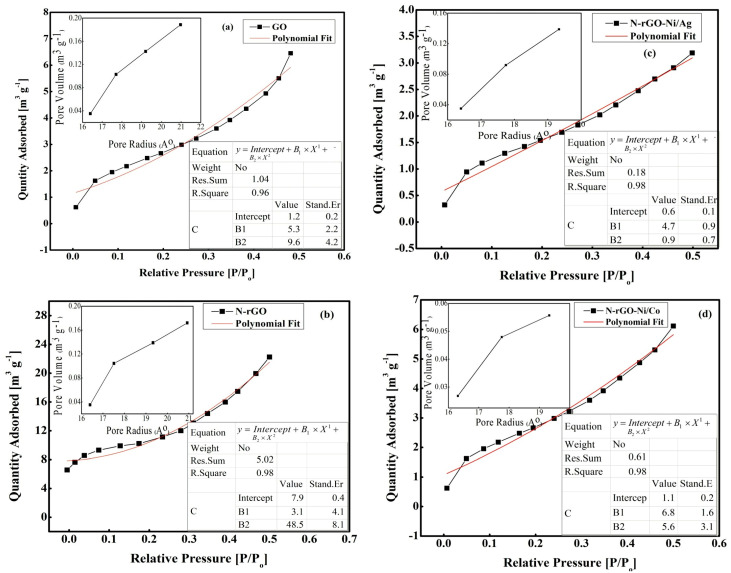
Brunauer–Emmet–Teller (BET) measurements of (**a**) GO, (**b**) N-rGO, (**c**) N-rGO-Ni/Ag and (**d**) N-rGO-Ni/Co.

Taking into consideration the BET, the adsorption isotherms of N_2_ were used to study the surface features of (a) GO, (b) N-rGO, (c) N-rGO-Ni/Ag and N-rGO-Ni/Co composites shown in Figure 5. The isotherms demonstrate a typical linear characteristic with correlation coefficient (R^2^) value ranging from 0.963–0.979. All the isotherms in Figure 5 represent the reversible type II isotherms having physisorption of nitrogen.

Regression analyses are usually used to model the functional relationship between two quantities. The best progression would be the one among different models, whose regression coefficient are closed to 1. Considering regression coefficient, we applied simple linear and polynomial regression models for the purpose to estimate the relationship between adsorbed quantities with respect to relative pressure. Using these models, the regression coefficients values were found better in polynomial fitting, that’s why polynomial fitting was applied. The reason for little deviations at low P/P_0_ values in the polynomial fit can be explained on the basis of pore filling contamination’ phenomenon where the small pores compatible with the molecular dimension of adsorbate filled first as compared to the larger pores, leading to the monolayer coverage. Once the monolayer is completed, the curvature is changed sharply, indicating the commencement of the multilayer adsorption [31]. An unrestricted monolayer-multilayer adsorption at high P/P_o_ might be accomplished.

The unique features of GO attract substantial research interest. Herein, GO (Figure 5a) and N-rGO (Figure 5b) show surface area of about 116.5 and 111.3 m^2^/g which is much smaller than the expected value (2630 m^2^/g) of graphene [32]. There are a number of reasons behind the facts proclaimed by Esmaeili et al. which may range from 2 to 1000 m^2^/g. Nevertheless, N-rGO-Ni/Ag (Figure 5c) shows better surface area of about 621.3 m^2^/g, while N-rGO-Ni/Co exhibit 528.3 m^2^/g which is satisfactory numbers in this regard. The pore volume and pore size distribution were found from Barret-Joyner-Halenda (BJH) while the specific surface area and correlation coefficient from Brunauer-Emmet-Teller (BET) method and are tabulated in Table 1.

### 3.6. X-ray Photoelectron Spectroscopy

**Figure 6:**
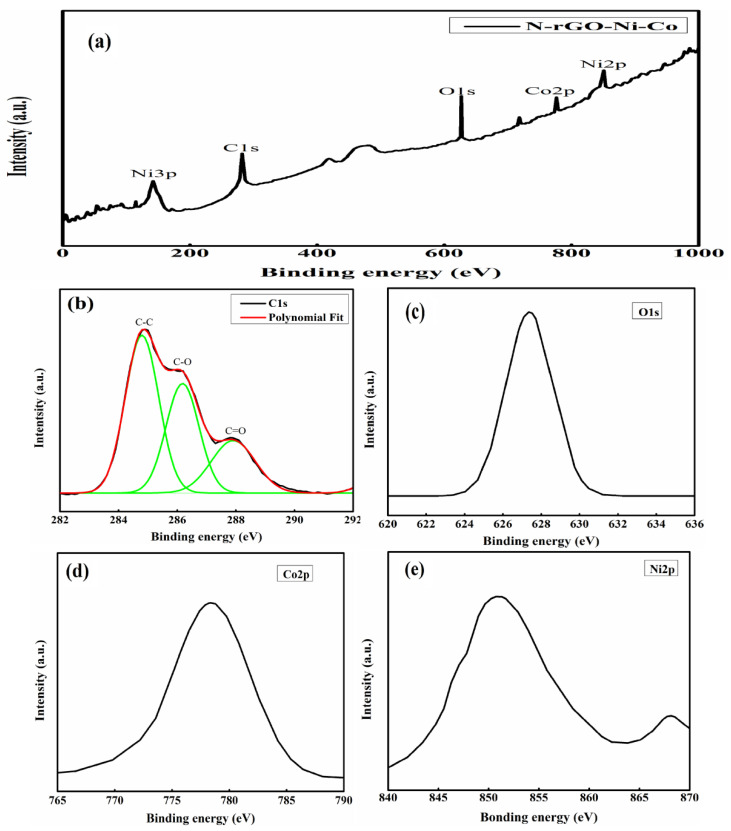
X-ray photoelectron spectroscopy of (**a**) N-rGO-Ni/Co (**b**) C1s, (**c**) O1s, (**d**) Co2p, and (**e**) Ni2p.

**Figure 7:**
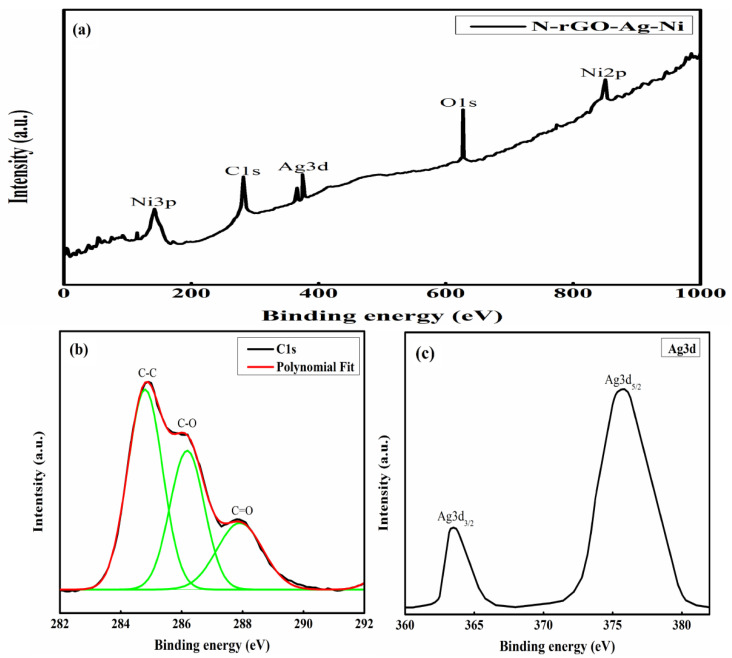
X-ray photoelectron spectroscopy of (**a**) N-rGO-Ni/Ag (**b**) C1s (**c**) Ag3d.

Figure 6 and Figure 7 display the full range XPS spectra of N-rGO-Ni/Co and N-rGO-Ni/Ag. N-rGO-Ni/Co survey in Figure 6a represent the spectral bands at 284.8, 627.3, 778.44, and 850.2 (main peak) eV which were indexed to C1s, O1s, Co2p and Ni2p, correspondingly. The binding energies of 778.44 and 850.2 eV are in accordance for Co2p3/2 and Ni2p3/2, which specify Co^+3^ and Ni^+2^ species in the composite system respectively [33]. Another small peak positioned at 860.34 eV in Figure 6e is represent Ni2p1/2. The C1s signal was deconvoluted into three climaxes (Gaussian and Lorentzian lines fitted (green)) in order to remove the broadening that resulted from instrumental factors and separate the “true” spectrum from the observed spectrum. The process leads to a new spectrum with the data altered by the deconvolution process. Each of these peaks represents the state which reflects the physical process involved in generating the original signal The peaks positioned at 284.8, 286.1, and 287.8 eV in C1s corresponds to the sp^2^ carbon, the epoxy, and the carboxyl functional groups, in that order [34,35]. The intensity of C–O band in C1s spectrum is fairly smaller than C–C manifesting reduction of GO. Inspecting the O1s spectrum in Figure 6c ascribe surface hydroxyl (OH) groups or even defects in the metal-graphene oxide lattice. The higher binding energy (627.3 eV) signifies the strong tie of Ni and Co within the composite. The peak for Co2p in Figure 6d corresponding to strong signals appeared at 778.44 eV is sign for deposition on the oxygen functionality in +3 oxidation state.

N-rGO-Ag/Ni survey in Figure 7a represent the spectral bands at 282.6, 627.3, and 850.2 (main peak) eV which represents C1s, O1s, and Ni2p, respectively. The detail description of C1s spectra is discussed in Figure 6b. The Ag3d core-level in Figure 7c has electronic states of Ag3d3/2 and Ag3d5/2 with binding energies of 363.6 and 375.7 eV are typical known for Ag [36]. Although the reduction of GO restored π-conjugation in the graphene, the residual oxygen functionalities in the N-rGO-Ni/Co and N-rGO-Ni/Ag were sufficient to retain colloidal stability in the aqueous medium. The additional peak about 418 eV obviously represents the nitrogen contents in the graphene framework. The XPS results are in close accords with FTIR and XRD.

### 3.7. ORR Activity

**Figure 8:**
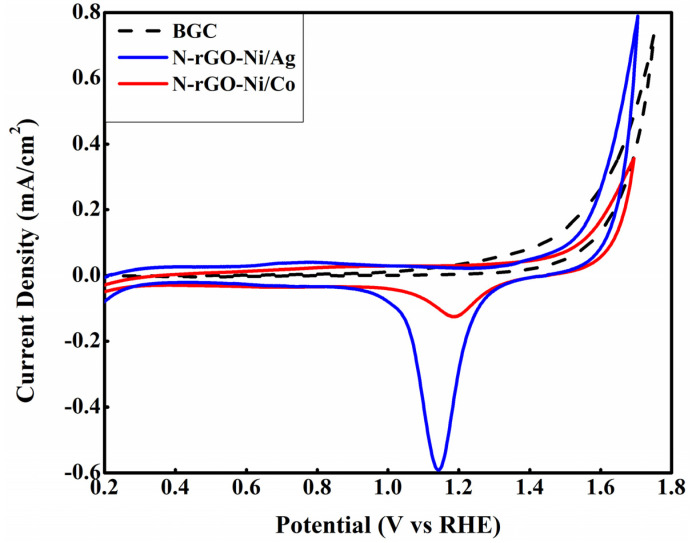
Cyclicvoltammetry curves for bare glassy carbon (BGC) electrode and modified GC with N-rGO-Ni/Ag and N-rGO-Ni/Co.

Figure 8 shows the cyclic voltammogram of bare glassy carbon electrode (BGC) N-rGO-Ni/Ag and N-rGO-Ni/Co composites in 0.5 M KOH, at scan rate 20 mV/s. The conventional BGC did not have any peak indicating no catalytic activity. While the sharp reduction peak for N-rGO-Ni/Ag suggests superior ORR activity. The ORR peak for N-rGO-Ni/Ag is located at 1.146 (V vs. RHE). Whereas, the ORR peak for N-rGO-Ni/Co is shifted more toward positive, which is about 1.186 (V vs. RHE) and is equivalent to the cathodic peak potential of Pt/C (1.185 V vs. RHE) catalyst [37].

**Figure 9:**
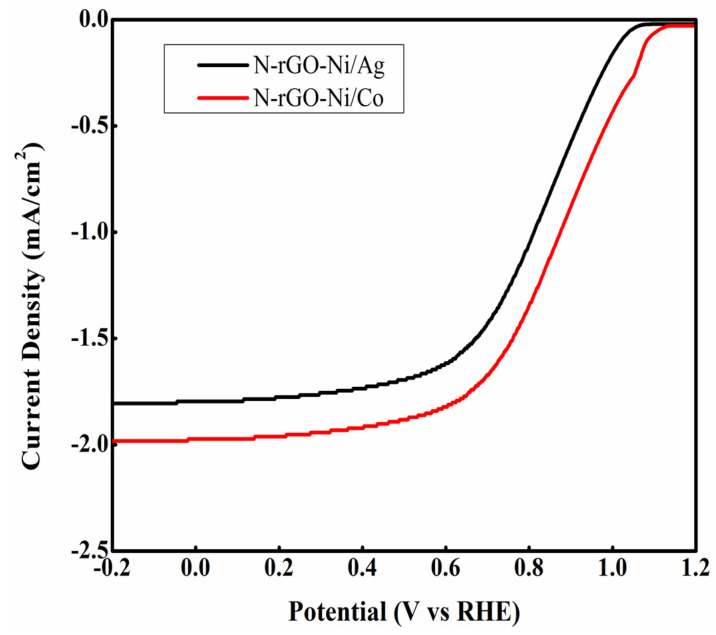
LSV measurement in O_2_-saturated 0.5 M KOH solution at 1200 rpm.

Figure 9 shows the LSV curves of N-rGO-Ni/Ag and N-rGO-Ni/Co at 1200 rpm. The ORR onset and half wave potential for N-rGO-Ni/Ag are observed at 1.096 and 1.046 (V vs. RHE) respectively. However, these values are shifted more toward positive potential (onset potential: 1.146, half wave potential 1.106 (V vs. RHE)) for N-rGO-Ni/Co which is in the vicinity of the commercial benchmark Pt/C catalyst [38].

**Figure 10:**
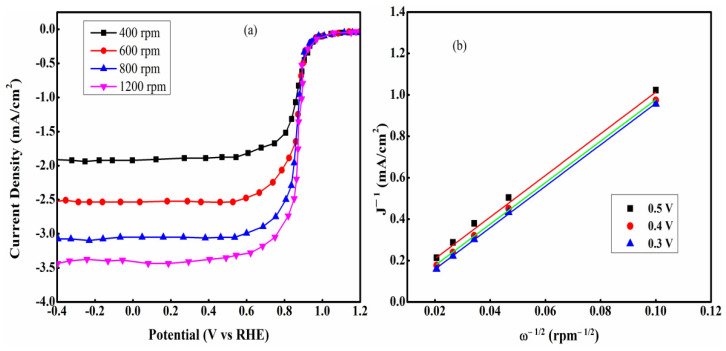
(**a**) Rotating disk voltammograms of N-rGO-Ni/Co inO_2_-saturated 0.5 M KOH at different rotation rates, (**b**) Koutecky-Levich plot of N-rGO-Ni/Co at electrode potential of 0.3, 0.4, and 0.5 V.

The N-rGO-Ni/Co catalyst was subjected to different rotation speed in O_2_ saturated 0.5 M KOH solution. It can be envisaged from Figure 10a that the mass transport is growing at the electrode surface with increasing rotation speed. The positive onset potential of +0.90 V and maximum current density of −3.44 mA cm^−2^ is observed at a rotation frequency of 1200 rpm. The number of electrons transferred (Figure 10b) to an oxygen molecule is calculated via Koutecky-Levich equation.

(2) $$\frac{1}{J}=\frac{\upsilon^{\frac{1}{6}}}{0.2{\mathrm{nFC}}_{0}D_{0}^{2/3}\omega^{\frac{1}{2}}}+\frac{1}{J_{K}}$$

where, J and J_K_ are the measured and kinetic-limiting current density respectively, *ω* is the angular velocity of the disc, n is the overall number of electrons transferred in the oxygen reduction, F is the Faraday constant (96,485 C/mol), C_0_ is the bulk concentration of oxygen (1.2 × 10^−6^ mol/cm^3^), D_0_ is the diffusion coefficient of oxygen in 0.5 M KOH (1.9 × 10^−5^ cm^2^/s), υ is the kinetic viscosity (0.01 cm^2^/s) and 0.2 is a constant that is valid when the rotation speed is expressed in rpm. The Koutecky-Levich plot of 1/J vs. *ω*^−1/2^ at a potential of +0.3, +0.4 and +0.5 V on the N-rGO-Ni/Co electrode (Figure 10b) shows good linearity (R^2^ = 0.996). The electron transfer number is about 3.7–3.8 at potential of 0.3 to 0.5 V, that suggesting a four-electron transferring pathway [39].

**Figure 11:**
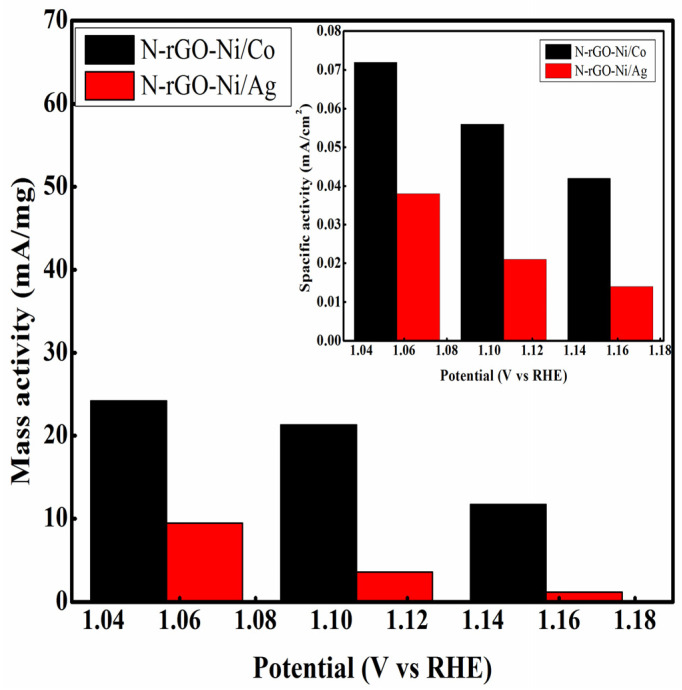
ORR mass activity and specific activity (inset) of N-rGO-Ni/Ag and N-rGO-Ni/Co at different potential.

The mass activity and Specific activity based on catalyst loading further verified the economic feasibility of the synthesized catalysts for commercial applications (Figure 11). The mass activities of the catalysts are measured by calculating the attained current density as a function of catalyst loading on unit area at known potential. The specific activity was also measured at a definite potential for unit electrochemical surface area of the catalysts. The mass activity of N-rGO-Ni/Co (mass loading 0.125 mg/cm^2^) attained a value of 24.5, 21.4 and 11.7 mA/mg at 1.056, 1.106 and 1.156 (V vs. RHE) respectively. These values are higher than that of N-rGO-Ni/Ag (1.056 V: 9.47 mA/mg, 1.106 V: 3.5 mA/mg, and 1.156 V: 1.3 mA/mg) and even the bench mark Pt/C catalyst (mass loading 0.1 mg/cm^2^ offered the mass activity of 23 mA/mg at 1.156 V). Considering the cost of platinum, the mass activity value of N-rGO-Ni/Co is very impressive and can be used as a state-of-the-art catalyst for ORR [38].

### 3.8. OER Activity

**Figure 12:**
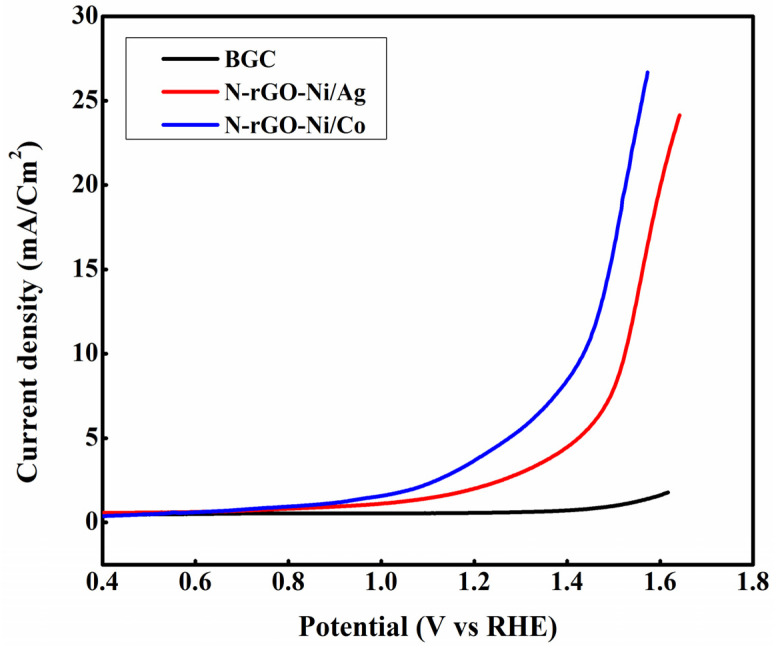
LSV curve of BGC, N-rGO-Ni/Ag, and N-rGO-Ni/Co for OER.

Figure 12 shows that N-rGO-Ni/Co catalyst has significantly higher anodic current and lower onset potential than that of the N-rGO-Ni/Ag and several time better that BGC. N-rGO-Ni/Co illustrate low onset potential at about 0.876 (V vs. RHE) and accomplished the current density of j = 1.26 mA/cm^2^, which is much better than that of the N-rGO-Ni/Ag which have onset potential 0.919 V and current density of j = 0.85 mA/cm^2^ [40,41].

**Figure 13:**
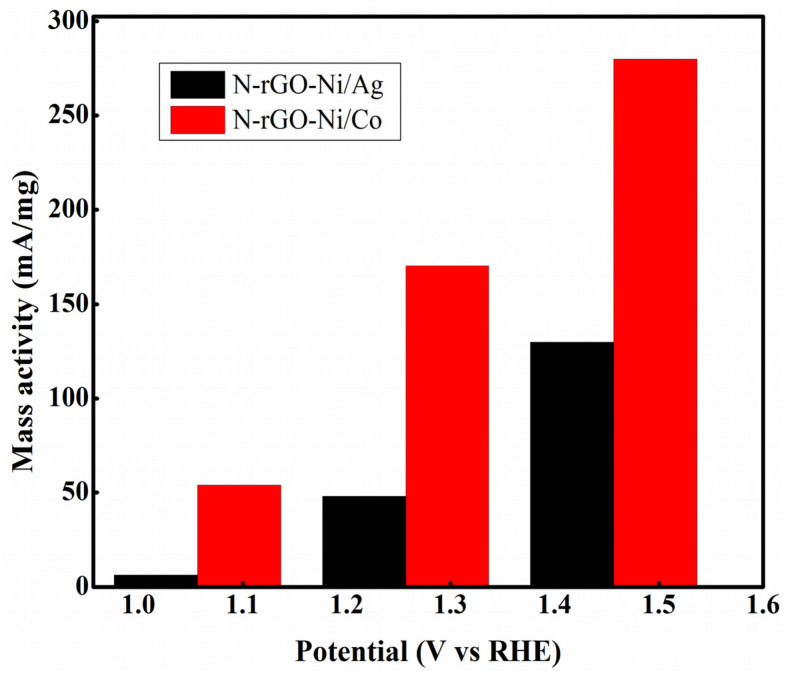
OER mass activity N-rGO-Ni/Ag and N-rGO-Ni/Co at different potential.

To assess the catalytic efficiency towards OER, it is noteworthy, to determine the mass activity of the catalysts which is shown in Figure 13. It is noted from the Figure that the mass activity of N-rGO-Ni/Co is 54.9 mA/mg at potential 1.056 (V vs. RHE) which is almost 8 times higher than N-rGO-Ni/Ag (6.2 mA/mg). The sweep rising in bar values are observed which are 170 and 280 at potential 1.256 (V vs. RHE) and 1.456 (V vs. RHE) respectively, as compared to N-rGO-Ni/Ag.

**Figure 14:**
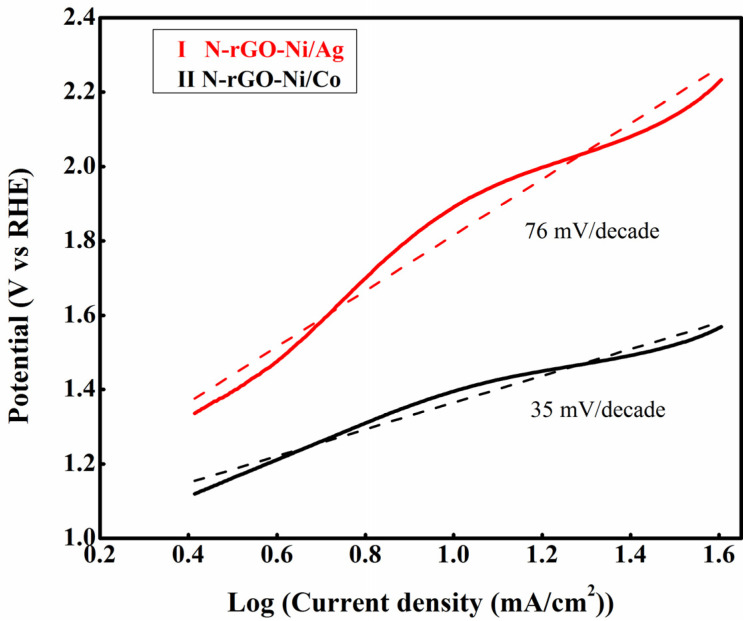
Tafel plots based on the LSV curve in 0.5 M KOH.

To evaluate the effectiveness of the catalysts in alkaline solutions (0.5 M KOH), Tafel plot was derived from the polarization curves using Tafel equation η = blog(j/j_0_), where η is the overpotential, b is the Tafel slope, j is the current density, and j_0_ is the exchange current density. The plot for N-rGO-Ni/Ag and N-rGO-Ni/Co (Figure 14) have slope values 76 and 35 mV/decade, respectively. The lower Tafel slope for N-rGO-Ni/Co corresponds to facile charge transfer and has a good chemical and electronic coupling. The small Tafel plot for N-rGO-Ni/Co correspond to favorable electrocatalytic activity towards OER kinetics compared to N- rGO-Ni/Ag. The OER parameters are listed in Table 2.

### 3.9. Chronoamperometric Stability

**Figure 15:**
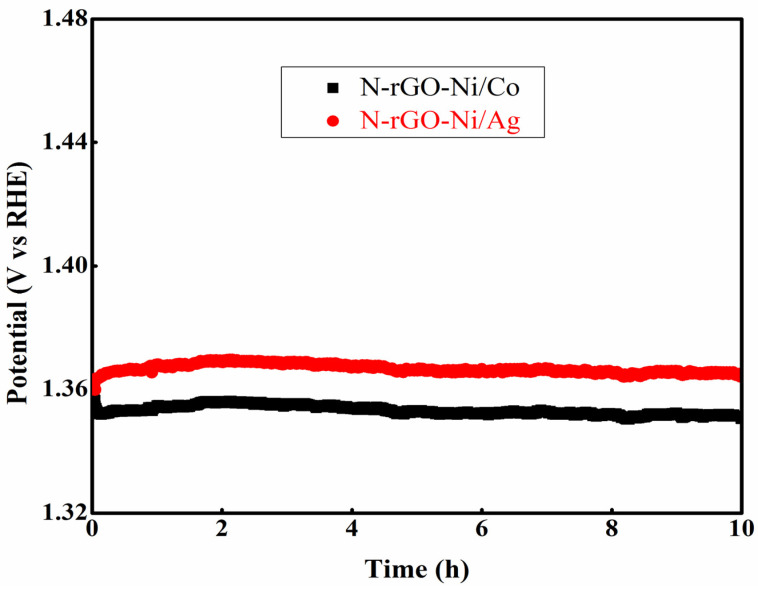
Chronoamperometric response recorded at applied potential of 1.36 V.

The catalytic stability was also studied by plotting potential as the oxygen reduction potential of N-rGO-Ni/Ag and N-rGO-Ni/Co as a function of time (Figure 15). Both catalysts reveal long term stability which reflects the importance of these catalysts for its practical application in alkaline media.

### 3.10. Bi-Functional Electrocatalytic Activity Measurement

**Figure 16:**
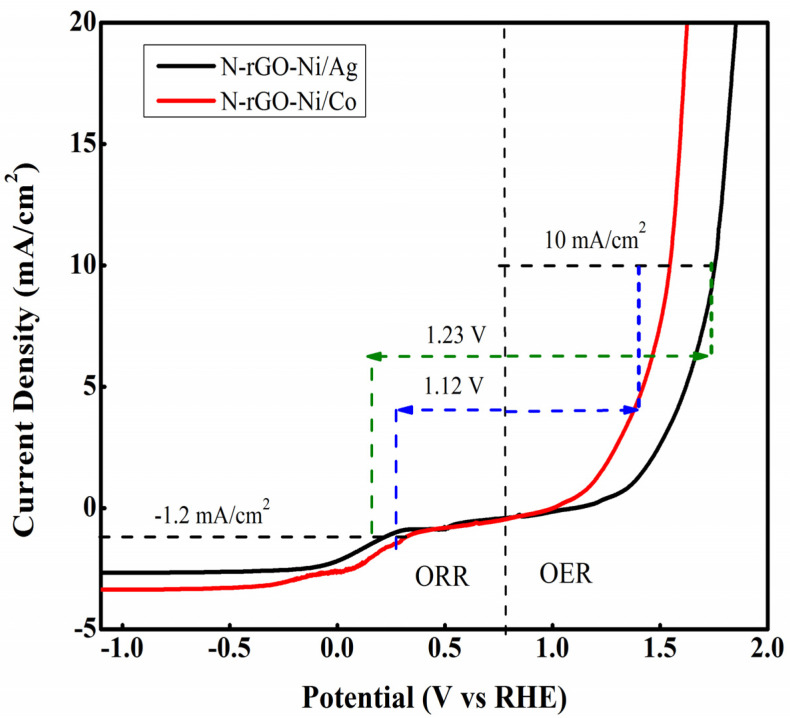
Oxygen electrode activities of both the catalysts within the range of potential for ORR and OER in O_2_ saturated 0.5 M KOH electrolyte at 1200 rpm.

The bi-functional electrocatalytic activity of the N-rGO-Ni/Ag and N-rGO-Ni/Co was calculated from the potential difference between OER and ORR such as ∆E = E_OER_(10 mA/cm^2^) − E_ORR_(−1.2 mA/cm^2^)_._ The benchmark current density has been considered 10 mA/cm^2^ and −1.2 mA/cm^2^ for OER and ORR, respectively. It is evident from the Figure 16 that the ∆E value has inverse relation with bi-functional catalytic activity towards OER and ORR. The potential difference ∆E for N-rGO-Ni/Ag and N-rGO-Ni/Co is 1.23 and 1.12 (V vs. RHE), respectively, which are reasonable values match to the other efficient electrocaytalysts listed in Table 3. However, N-rGO-Ni/Co has indeed better electrocatalytic act in response than that of N-rGO-Ni/Ag.

### 3.11. Electrochemical Impedance Spectroscopic (EIS) Analysis

**Figure 17:**
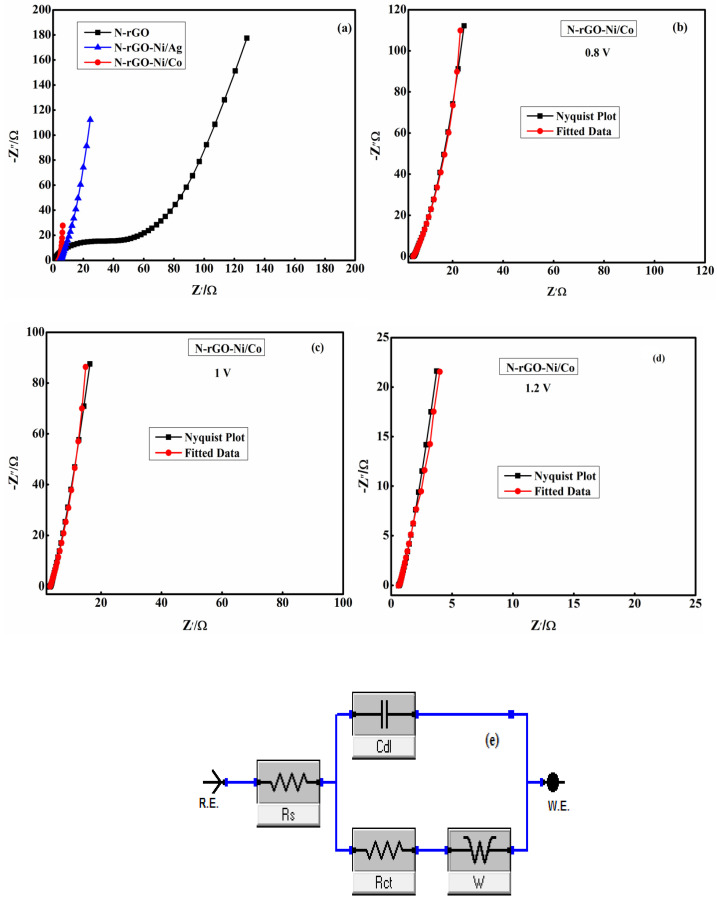
Nyquist plots for (**a**) N-rGO, N-rGO-Ni/Co and N-rGO-Ni/Ag (**b**) N-rGO-Ni/Co at 0.8 V, (**c**) 1 V, (**d**) 1.2 V and (**e**) proposed equivalent circuit.

Figure 17 shows the faradic charge transfer resistance (R_ct_) and internal resistance (R_s_) of the above mentioned materials which were analyzed via EIS analysis in correspondence with Nyquist plots at different voltages. The semicircles at high and low frequency range in the Nyquist plots are accredited to faradic charge transfer resistance R_ct_ and the internal resistance R_s,_ respectively [43]. The inclined line at low frequency area portrays the transfer and diffusion of the electrons amongst the functional groups of graphene and metal ions. The shortness and closeness of the inclined line toward *y*-axis is manifesting the superior electrocatalytic behavior of the materials [44,45].

Accordingly, N-rGO-Ni/Co and N-rGO-Ni/Ag showed lower R_ct_ value than N-rGO, suggesting a lower frequency and higher charge transport efficiency for N-rGO-Ni/Co and N-rGO-Ni/Ag. This behavior entails superior electrocatalytic activity by boosting the electron transferring at the electrode interface [46]. Nevertheless, this tendency is more prominent in N-rGO-Ni/Co than N-rGO-Ni/Ag.

Based on the aforementioned considerations, N-rGO-Ni/Co sample was further studied at different voltages and the Nyquist plots were fitted with the proposed equivalent circuit model using Echem Analyst software. Where the R.E, W.E, R_s_, R_ct_, W, and a represent reference electrode, working electrode, internal or solution resistance, charge transfer resistance, Warburg impedance and electrical double layer capacitance in that order. We mainly emphasized on the R_s_ and R_ct_ of the electrode in this study. The R_ct_ value is significantly decreased from 2.978 Ω at 0.8 V to 1.200 Ω at 1.2 V. These values highly impact the inclined line which is progressively shifted towards the *y*-axis with the increase in potential. The Nyquist plots suggests the enhance electrocatalytic tendency of N-rGO-Ni/Co than N-rGO-Ni/Ag. The different impedance parameters elucidated from proposed equivalent model are listed in Table 4.

## 4. Conclusions

The composites of N-doped reduced Graphene oxide (N-rGO) with Ni/Ag and Ni/Co(non-precious transition metals) were successfully synthesized using reflux condensation reduction route. Different characteristics such as functional group determination, crystallinity, surface morphology, pore volume and pore size distribution of the synthesized samples were characterized and analyzed through FTIR, XRD, SEM, TEM, BET/BJH and XPS. The complexation of different functionalities of GO with Ni/Ag and Ni/Co appears in the form of overlapping of the FTIR, and XPS spectra. The XPS analysis further verified the electronic states with characteristic binding energies of different species where the binding energies of 778.4 and 850.2 eV were found to be in accordance with Co2p3/2 and Ni2p3/2 specifying Co^+3^ and Ni^+2^ species in the composite system. XRD analysis revealed the crystalline nature with average crystallite size of about 4.593 nm as calculated by using Scherer’s equation. A surface morphology with flaky texture morphology with several cavities and carbon layers with inter layer distance of about 0.35 nm was observed with the help of SEM and TEM analysis. The composites were found to be suitable for bi-functional ORR and OER capability. They exhibited low charge transfer resistance and better stability (10 h) as verified by LSV, EIS and chronamperometric analysis. However, the overall performance of N-rGO-Ni/Co composites was better then N-rGO-Ni/Ag composites that might be attributed to better interaction and compatibility of Ni with Co. Considering the better properties of N-rGO-Ni/Co (1.146 onset potential, 1.106 half wave potential, Tafel value of 35 mV/decade, bifunctional activity of 1.12 V, mass activity of 24.5 for ORR and 54.9 for OER) it can be utilized as a good alternative of nobel metals (Pt, Pd, Ir) based nanocomposites in fuel cell technologies.

## Figures and Tables

**Table 1:** BET and BJH measurements of GO, N-rGO, and its composites.

Sample	Pore Volume	Pore Size	Surface Area	Correlation
	(m^3^/g) (BJH)	(A^o^) (BJH)	(m^2^/g) (BET)	Coefficient (R^2^)
GO	0.02	16.4	116.5	0.96
N-rGO	0.02	16.4	111.3	0.98
N-rGO-Ni/Ag	0.14	17.7	621.3	0.98
N-rGO-Ni/Co	0.12	16.4	528.2	0.98

**Table 2:** OER parameters of N-rGO-Ag-Ni and N-rGO-Ni-Co.

Sample	Onset Potential	Tafel Slope	Mass Activity
	(V vs. RHE)	(mV/Decade)	(mA/mg)
N-rGO-Ni/Co	1.063	35	54.9
N-rGO-Ni/Ag	1.063	76	6.2

**Table 3:** Bi-functionality of N-rGO-Ni/Ag and N-rGO-Ni/Co catalyst compared with other catalysts reported in literature.

Catalysts	ORR (V vs. RHE)		OER (V vs. RHE)		∆E E_j=10_ − E_j=−1.2_	Ref.
	Onset Pot.	E_j^2^=−1mA/cm_	Onset Pot.	E_j^2^=10mA/cm_	(V vs. RHE)	
N-rGO-Ni/Ag	1.146	0.28	1.063	1.52	1.23	Present work
N-rGO-Ni/Co	1.146	0.17	1.063	1.61	1.12	Present work
NiCo_2_O_4_/G	1.126	0.62	1.796	1.75	1.13	[42]
20 wt% Pt/C	1.226	0.84	1.956	1.83	0.99	[34]

**Table 4:** Parameters derived from Nyquist plots of N-rGO-Ni/Co at different voltages.

Voltage (V)	R_ct_ (Ω/cm^2^)	R_s_ (Ω/cm^2^)
0.8	2.978	6.44 × 10^−1^
1	1.739	7.39 × 10^−1^
1.2	1.200	8.84 × 10^−1^

## Data Availability

Data is available on request from the first author.
